# The Role of Genetics in Preterm Birth

**DOI:** 10.1007/s43032-023-01287-9

**Published:** 2023-07-14

**Authors:** Elyse C. Mead, Carol A. Wang, Jason Phung, Joanna YX Fu, Scott M. Williams, Mario Merialdi, Bo Jacobsson, Stephen Lye, Ramkumar Menon, Craig E. Pennell

**Affiliations:** 1 School of Medicine and Public Health, University of Newcastle, Newcastle, NSW 2308 Australia; 2 Hunter Medical Research Institute, Newcastle, NSW 2305 Australia; 3 Department of Maternity and Gynaecology, John Hunter Hospital, Newcastle, NSW 2305 Australia; 4 Department of Population and Quantitative Health Sciences, School of Medicine, Case Western Reserve University, Cleveland, OH USA; 5 Maternal Newborn Health Innovations, Geneva, PBC Switzerland; 6 Department of Obstetrics and Gynaecology, Institute of Clinical Science, Sahlgrenska Academy, University of Gothenburg, Gothenburg, Sweden; 7 Department of Obstetrics and Gynaecology, Region Västra Götaland, Sahlgrenska University Hospital, Gothenburg, Sweden; 8 Department of Genetics and Bioinformatics, Domain of Health Data and Digitalization, Institute of Public Health, Oslo, Norway; 9 Lunenfeld Tanenbaum Research Institute, Toronto, Ontario Canada; 10 Department of Obstetrics and Gynecology, Division of Basic Science and Translational Research, University of Texas Medical Branch, Galveston, TX USA

**Keywords:** Preterm birth, Gestational duration, Genomics, Transcriptomics, Epigenetics, Multiple “-omics” analyses

## Abstract

Preterm birth (PTB), defined as the birth of a child before 37 completed weeks gestation, affects approximately 11% of live births and is the leading cause of death in children under 5 years. PTB is a complex disease with multiple risk factors including genetic variation. Much research has aimed to establish the biological mechanisms underlying PTB often through identification of genetic markers for PTB risk. The objective of this review is to present a comprehensive and updated summary of the published data relating to the field of PTB genetics. A literature search in PubMed was conducted and English studies related to PTB genetics were included. Genetic studies have identified genes within inflammatory, immunological, tissue remodeling, endocrine, metabolic, and vascular pathways that may be involved in PTB. However, a substantial proportion of published data have been largely inconclusive and multiple studies had limited power to detect associations. On the contrary, a few large hypothesis-free approaches have identified and replicated multiple novel variants associated with PTB in different cohorts. Overall, attempts to predict PTB using single “-omics” datasets including genomic, transcriptomic, and epigenomic biomarkers have been mostly unsuccessful and have failed to translate to the clinical setting. Integration of data from multiple “-omics” datasets has yielded the most promising results.

**Supplementary Information:**

The online version contains supplementary material available at 10.1007/s43032-023-01287-9.

## Introduction

Preterm birth (PTB) (defined as the birth of a child before 37 completed weeks gestation) is the leading cause of death and disability in children under 5 years of age worldwide [1, 2]. Whilst PTB is becoming a preventable condition for a very small subset of women [3–9], global rates of PTB continue to rise [10, 11]. Recent estimates report that PTB affects approximately 11% of all livebirths, or approximately 15 million PTB per year [11]. PTB has substantial short- and long-term sequelae related to physical and neurocognitive development [12, 13]. Short-term, PTB is associated with early mortality [14–16]. Long-term complications of PTB include early development of adult-onset diseases like certain cancers, cardiovascular disease, insulin resistance, chronic kidney disease, obesity, and neurodevelopmental and social disability [17, 18]. Multiple risk factors for PTB have been identified including environmental factors such as demographic and lifestyle risk factors, maternal medical disorders, and antenatal risk factors (Table 1). One of the strongest risk factors, and thus greatest predictors, for PTB is a prior history of preterm delivery [19, 20], indicating that there may be genetic predispositions to PTB. Since the first draft of the human genome sequence was completed in 2001 [21], there has been an increase in information regarding the genetics of diseases, including PTB. Significant advances in technology have occurred within the last two decades that have increased the ability to obtain genetic data, providing the opportunity to address challenges in preventing and managing diseases. Studies have been conducted to improve the understanding of biological mechanisms underpinning PTB and translate research findings into a clinical setting. The development of prediction tools that stratify care to provide targeted and personalized prevention of PTB has been a major focus in many PTB-related genetic studies.

**Table 1:** Maternal risk factors for preterm birth [19, 20, 22–29]

Maternal demographic & lifestyle risk factors	Maternal medical disorders & pregnancy risk factors
Cigarette smoking Illicit drug use Alcohol intake Psychosocial stress Exposure to environmental pollutants and xenobiotics Low socioeconomic and educational status Low (<18 years) or high (>40years) maternal age Low (< 18) or high BMI (> 30) Single marital status Aboriginal or Torres Strait Islander ethnicity African American ethnicity Short inter-pregnancy interval Malnutrition: • Low pre-pregnancy body mass index • Obesity • Deficits in serum folate, iron, zinc, omega 3, and vitamin D	Medical conditions including thyroid disease, hypertension, diabetes, and asthma In vitro fertilization Uterine malformations Intrauterine infections Urogenital and other systemic infections Intra-amniotic infection Cervical insufficiency occurring from the following: • Large loop excision of the transformation zone • Altered cervical composition due to inflammation • Inherited cervical malformations Abnormal placentation Antepartum hemorrhage Preterm prelabor rupture of the membranes Multifetal pregnancies

## Preterm Birth Phenotype

**Fig. 1:**
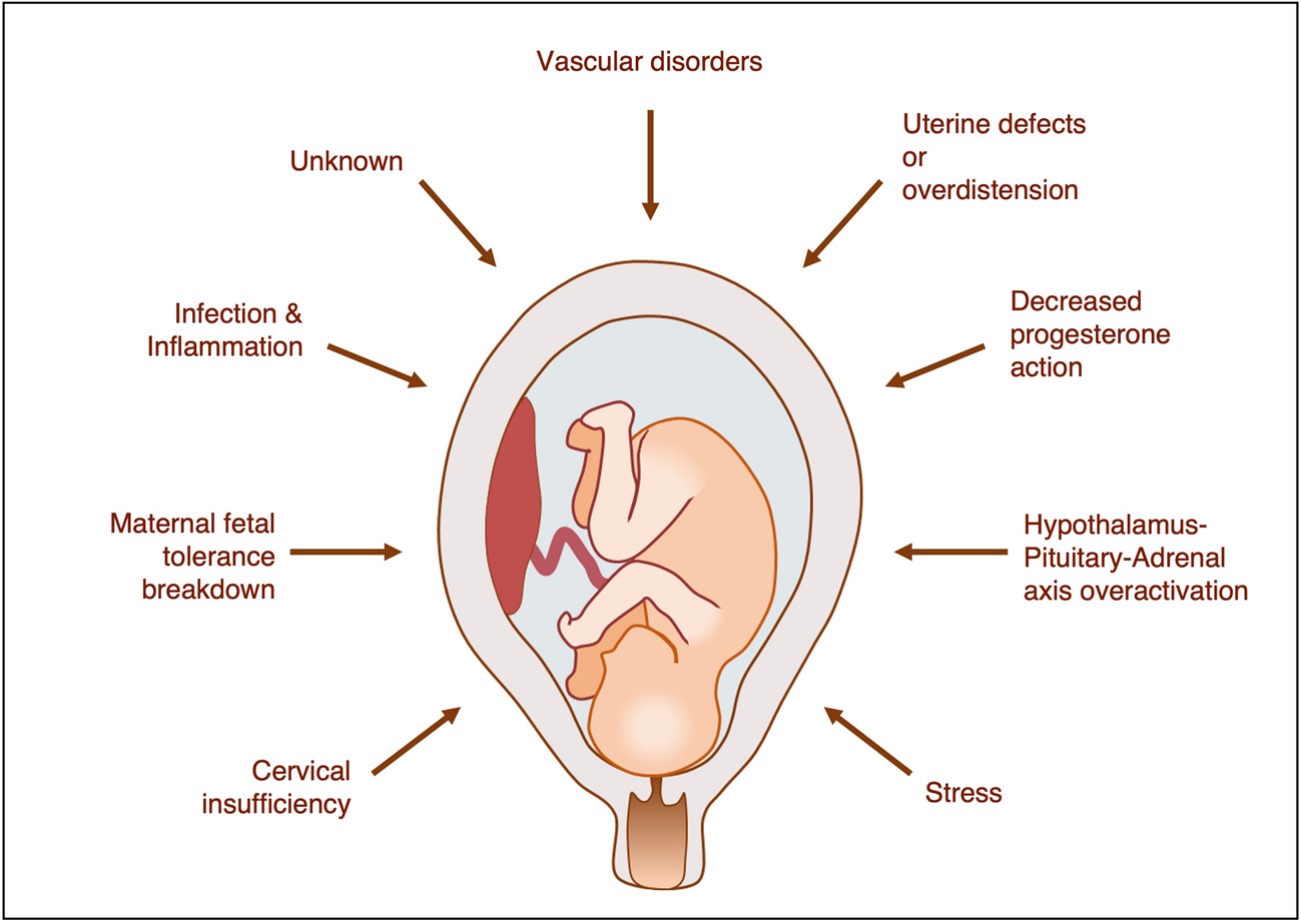
Mechanisms proposed to underpin spontaneous preterm birth. Figure adapted from [30]

**Fig. 2:**
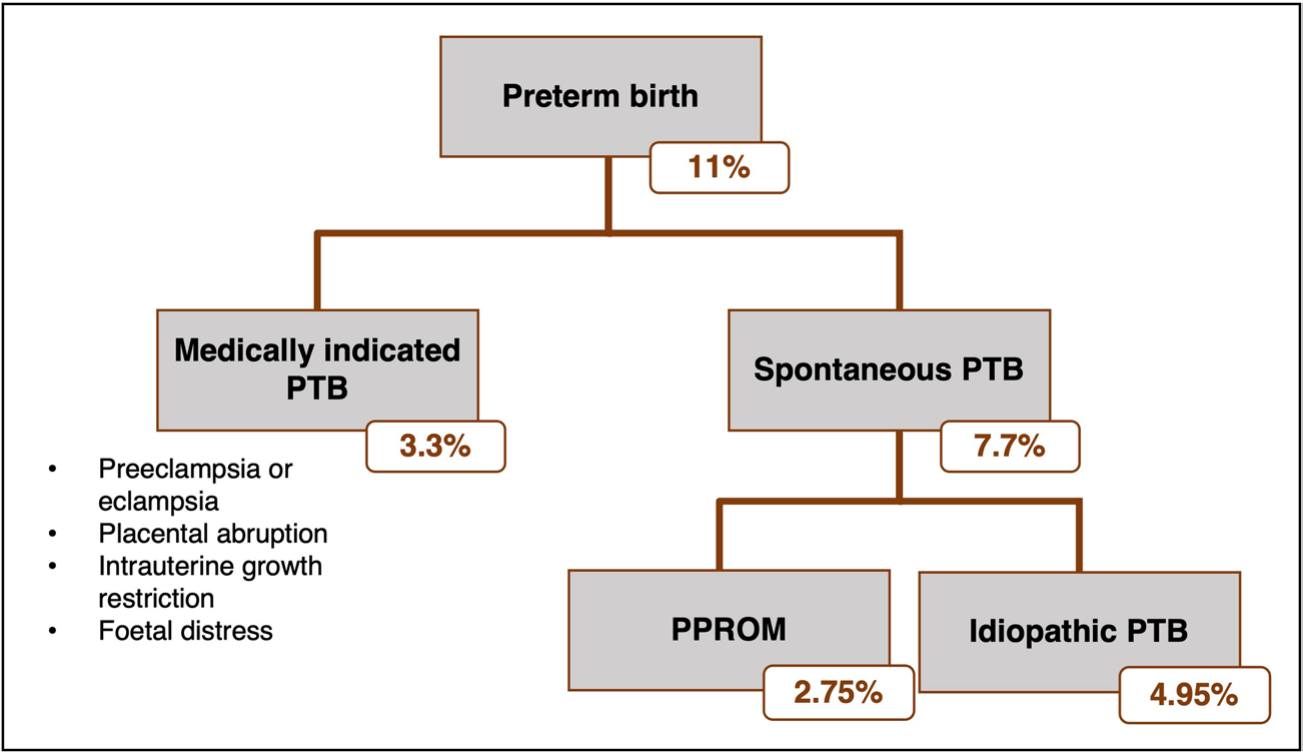
Preterm birth (PTB) phenotypes. PTB accounts for approximately 11% of all livebirths and can be subcategorized into medically indicated PTB and spontaneous PTB (sPTB). sPTB includes both preterm prelabor rupture of membranes (PPROM) and idiopathic PTB. PTB can be medically indicated for several reasons involving both mother and baby. Estimates are based off data published by Chawanpaiboon et al. and Morken et al. [11, 31]

Investigating the genetics of PTB is challenging because the PTB population is highly heterogenous. Multiple mechanisms have been implicated in PTB development including infection and inflammation, excess activation of the hypothalamic-pituitary-adrenal axis, uterine defects or over-distention, stress, cervical disease, vascular disease, maternal-fetal tolerance disruption, and decreased progesterone action (Fig. 1) [30]. Independent of these various risk factors, PTB can be grouped into three broad clinical phenotypes: medically indicated PTB, idiopathic PTB (iPTB), and preterm prelabor rupture of membranes (PPROM). The latter two are often grouped as “spontaneous PTB (sPTB)” because labor occurs spontaneously in both cases (Fig. 2) [31]. PTB can also be subcategorized based on gestational age at delivery: for example, the World Health Organization categorizes PTB as extreme PTB (<28 weeks’ gestation), very preterm (28 to <32 weeks’ gestation), and moderate or late PTB (32 to <37 weeks’ gestation) [32]. One approach to study the genetics of PTB is to aggregate all sub-phenotypes into a single broadly phenotyped cohort. If the genetic mechanisms underpinning medically indicated PTB and sPTB are shared, combining sub-phenotypes may increase statistical power [33]. An alternative approach is to stratify by the diverse clinical conditions of PTB. Given the complex and heterogenous nature of PTB, testing in more homogenous cohorts may increase the sensitivity to detect genetic associations, even if they are only limited to specific risk groups [34].

## Heritability of Preterm Birth

The heritability of PTB has been assessed through large intergenerational studies, as well as in studies of monozygotic twins and siblings. There is a 20% increased risk of PTB if a mother was born preterm herself; this risk is inversely correlated to the mother’s gestational age at birth [19, 35]. Furthermore, women with sisters who delivered preterm babies are at an 80% increased risk of delivering preterm themselves [20, 36]. Twin studies have also suggested that genetics account for 17–36% of PTB risk [37, 38]. In attempting to understand the heritability of PTB, both maternal and fetal genetics have been assessed. It has been suggested that maternal genetics are responsible for 22.8% of the variations in gestational age in spontaneous births, with fetal genetics responsible for 12.7% of the variation [39]. The rates of PTB have also been consistently higher among African Americans, even after adjustment for socioeconomic confounders [35, 40–44]. The ethnic disparities, in conjunction with findings from twin and heritability studies, have driven researchers to further explore the genetics underpinning this disease.

## Approaches to Investigate the Genetic Basis of Preterm Birth

There are three distinct observational study designs that are commonly used to investigate the genetic basis of diseases including PTB: cohort studies, case-control studies, and family-based studies [34]. Cohort studies define a population either retrospectively or prospectively based on exposure [45]. An advantage of cohort studies is that they allow gestational age to be considered as a continuous outcome. In contrast, case-control studies retrospectively define a population based on outcome and dichotomize the cohort to assess for, in this case, genetic variants present in cases versus controls [45]. Family-based studies, such as case-parent trio analyses, utilize relatives as controls to overcome population stratification. In PTB genetic studies, family-based approaches allow for the genetic contributions of both the mother and baby to be assessed [34, 46].

Employing these broad study designs, many approaches to investigate the genetics of PTB have been established including large intergenerational studies, twin studies, family-based linkage studies, candidate gene studies, genome-wide association studies (GWAS), whole-exome sequencing (WES) and whole genome sequencing (WGS) analyses, copy number variant (CNV) analyses, and mitochondrial genetic studies [47, 48]. Research has also been conducted to explore gene-gene interactions, gene-environment interactions, epigenetics modifications, and transcriptomic changes associated with PTB [49]. Some studies have investigated the biological consequences of genetic polymorphisms by assessing the proteome and metabolome of PTB cases [50, 51]. Recently, studies have also conducted multiple “-omics” analyses to investigate the complex disease of PTB [52].

**Fig. 3:**
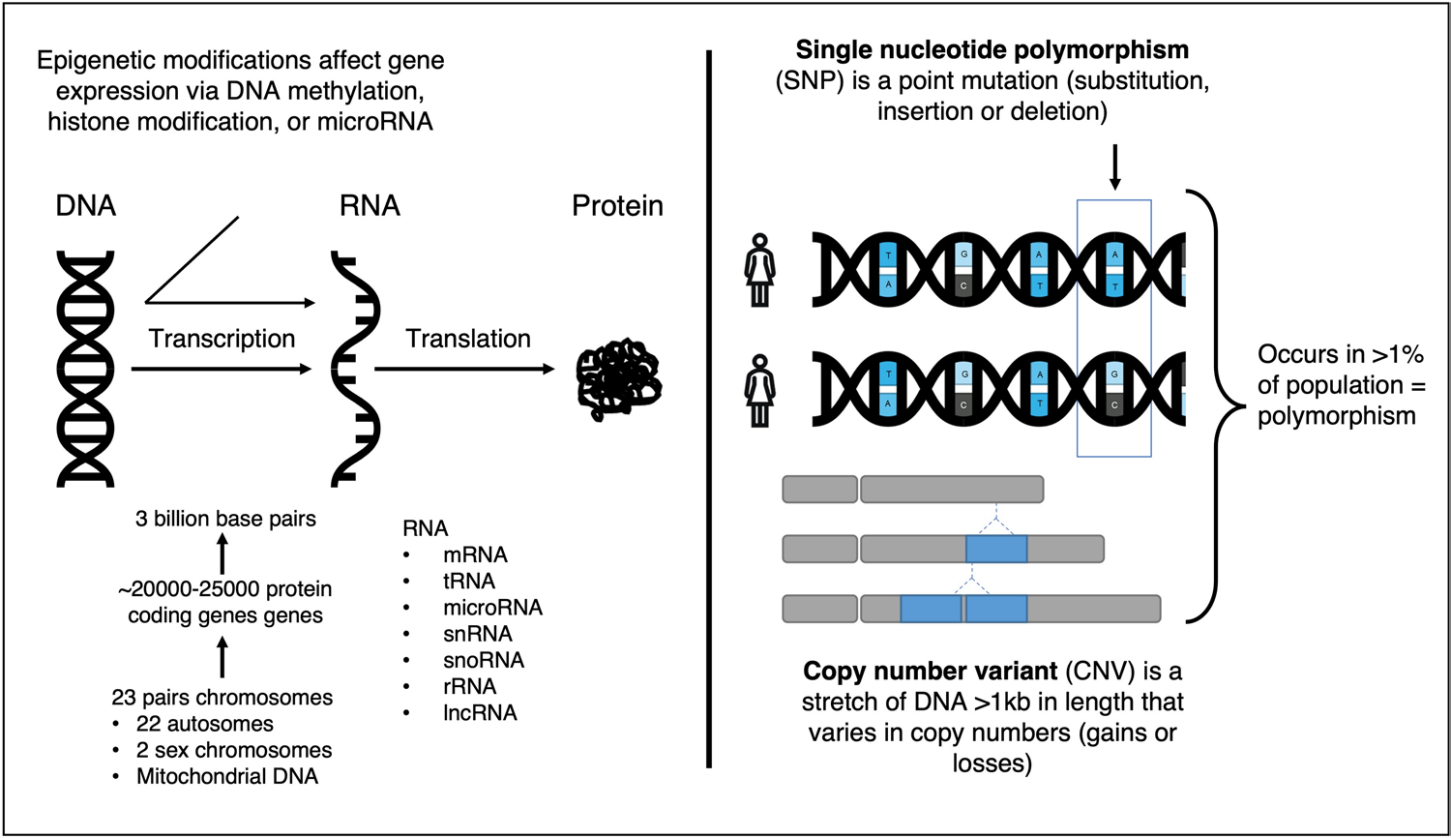
An overview of basic genetic concepts. The left-hand side of the figure shows a simplified depiction of DNA transcription to protein production. The right-hand side of the figure summarizes the definition of single nucleotide polymorphisms (SNPs) and copy number variants (CNV)

This review will focus on candidate gene studies, GWAS, WES and WGS analyses, CNV analyses, and de novo mutation analyses, as well as PTB-related transcriptomic and epigenomic studies. Refer to Table 2 and Fig. 3 for definitions and descriptions of the genetic variants and study designs mentioned in this review. We aim to present a comprehensive and updated summary of the published data relating to this field of research reviewing the literature arising from PubMed searches.

**Table 2:** Definitions and descriptions of genetic variants and study methodologies [53–62]

Genetic variant	Description and comments
Single nucleotide variant (SNV)	A genetic point mutation. SNVs have potential to cause downstream effects on gene function.
Single nucleotide polymorphism (SNP)	SNVs that occur in >1% of the population are SNPs. There are currently 720 million known SNPs in the human genome (as per NCBI dbSNP database build 155).
Copy number variant (CNV)	A stretch of DNA greater than 1kilobase in length that varies in copy numbers. These can be copy number losses (deletions or null genotypes) or gains (insertions or duplications) and can range from being simple in structure to involving complete gains or losses of genetic sequences. CNV have the potential to affect the function of multiple genes simultaneously.
De novo mutations	Rare non-inherited genetic variants and have potential to be pathogenic.
Study methodology	Description and comments
Candidate gene study	Assess SNPs known to be involved in regulating the function of pre-defined candidate genes and test for a statistical correlation with the disease of interest.
Genome-wide Association Study (GWAS)	Utilize a genome-wide and hypothesis-free approach. DNA is genotyped on an array to assess hundreds of thousands of known index SNPs within large cohorts and highlight genetic variants that may be associated with a disease or trait of focus. The index SNPs on GWAS arrays represents all SNPs in high linkage disequilibrium (LD) with the index SNP. As a result, the associations identified in GWAS are indirect as the casual SNP is often not the SNP identified in the GWAS, but rather a SNP in high LD.
Whole-exome sequencing (WES) & whole genome sequencing (WGS)	WGS sequences the entire DNA of the individual genome. WES is a targeted version of WGS that only sequences the exomes (protein coding regions) of the genome. These studies can detect rare or *de novo* variants associated with disease.
Transcriptomics	Analyses RNA transcripts, their expression, function, and degradation. RNA transcripts include both coding and non-coding RNA. Messenger RNA (mRNA) is a coding RNA. Non-coding RNA have no protein-coding potential, however, can be involved in post-translational regulation and other mechanisms that affect gene expression. An example of short non-coding RNAs (40-200 nucleotides in length) is microRNA (miRNAs). RNAs >200 nucleotides in length include ribosomal RNA (rRNA) and long non-coding RNA (lncRNA). The quantity of RNA transcripts is measured via microarrays or RNA sequencing for a given gene in specific tissues, providing a direct indication of gene transcription levels.
Epigenetics	Assess heritable alterations in the DNA that are not due to sequence changes in the genome. Epigenetic modifications occur primarily through DNA methylation, histone modification and miRNA, and are influenced by combined environmental exposures and genetic predispositions. Gene expression is regulated at both transcription events (DNA methylation and histone modification) and translation events (miRNA). Epigenetic changes often affect chromatin structure and accessibility which affect gene expression and protein transcription. Methylation often occurs at cytosines followed by guanosine residuals (CpG sites). DNA methylation can be studied using DNA methylation arrays and high-throughput sequencing. Chromatin accessibility assay ATAC-seq is used to profile histone modification.

## Preterm Birth Genetics

### Candidate Gene Studies

The candidate gene approach has been applied in many studies that aim to understand the mechanisms underlying PTB and identify potential biomarkers of prematurity. Candidate gene studies have identified over 750 SNPs within more than 240 genes in both mothers and infants of varying ethnicities that may be associated with PTB or gestational duration at birth (Online resource 2). This review aims to broadly discuss and summarize the findings from the large number of candidate gene studies published. These studies have implicated genes involved in tissue remodeling, vascular, endothelial, metabolic, inflammatory, and immune processes.

#### Inflammatory and Immunological Pathway-Related Polymorphisms

Inflammation is a biological response of the immune as well as nonimmune system [63], and inflammatory responses are tightly regulated during pregnancy [64, 65]. It is thought that labor is partly initiated by a switch from a balanced inflammatory state to a pro-inflammatory state [66]. Genetic polymorphisms that play a role in immunological responses during pregnancy could potentially result in overwhelming inflammatory responses and subsequently impact gestational duration. Candidate gene studies have extensively analyzed genomic loci involved in these processes, with a prominent focus on the innate immune system.

Polymorphisms within genes involved in pathogen recognition have been associated with both early and late PTB. Specifically, toll-like receptor 1 (*TLR 1*), toll-like receptor 2 (*TLR 2*), and toll-like receptor 7 (*TLR 7*) gene polymorphisms have exhibited associations with PTB when analyzing maternal samples [67–69]. Studies analyzing infant samples have highlighted similar results [68, 70].

Inflammation can be activated by both pathogen recognition and the recognition of tissue damage. It is an integral component of the innate immune system and is produced by eicosanoids and cytokines that are released from affected cells [63]. Genes encoding both pro- and anti-inflammatory cytokines, particularly interleukins, have been investigated in several candidate gene studies. Polymorphisms within genes encoding pro-inflammatory interleukins have been associated with PTB in mothers and infants [68, 71–75]. Several studies have also identified SNPs associated with PTB in genes encoding anti-inflammatory cytokines like interleukin 10 (*IL10*) and interleukin 13 (*IL13*) genes [67, 76–78]. Furthermore, genes encoding interleukin receptors and co-receptors involved in immunomodulation have been highlighted as potential genes involved in PTB [69, 79–81]. In addition to interleukins, cytokines such as tumor necrosis factor-α (TNFα), a pro-inflammatory cytokine, have been implicated in candidate gene studies that have investigated polymorphisms within encoding genes [71, 82–85].

Polymorphisms within genes involved in complement activation and regulation, as well as complement-opsonized pathogen clearance, have been associated with PTB [86, 87]. Furthermore, genes involved in innate immune cell functions such as cell migration and receptor function have been highlighted as potential risk genes [88, 89].

Genetic polymorphisms within genes involved in B and T cell activation, migration, and proliferation were implicated in those with preterm delivery [76, 80]. Furthermore, maternal polymorphisms within genes encoding proteins involved in immunoglobulin synthesis and function have been identified as potential risks for PTB [90–92].

#### Tissue Remodeling Pathway-Related Polymorphisms

Extensive tissue remodeling occurs throughout pregnancy and labor; the uterus gains smooth muscle and connective tissue, and prior to labor, the cervix softens [93]. Genes involved in tissue remodeling and hormones involved in both maintaining pregnancy and parturition have been analyzed. SNPs within genes involved in collagen biosynthesis and the synthesis and inhibition of matrix metalloproteases have exhibited associations with PTB [68, 94–96]. Polymorphisms within genes encoding fibroblast growth factors 1 and 4, both involved in embryonic development, morphogenesis, cell growth, and angiogenesis, have demonstrated associations with sPTB in maternal and infant samples [68, 87, 97]. Genes encoding parturition-related hormones including progesterone, follicle-stimulating hormone, corticotrophin-releasing hormone, and relaxin have also been implicated in candidate genes studies [98–104]. Kim et al. (2013) analyzed approximately 650 case-parents triads; maternal genetic effect analyses identified SNPs within leucyl and cysteinyl aminopeptidase gene (*LNPEP*) associated with prematurity*. LNPEP* encodes an enzyme responsible for the degradation of hormones vital for maintaining pregnancy [105].

#### Metabolic and Biosynthetic Pathway-Related Polymorphisms

Sustaining metabolic homeostasis is vital for embryonic development and survival. Polymorphisms within genes responsible for the biosynthesis and metabolism of fatty acids, lipoproteins, triglycerides, and cholesterol have been associated with PTB in women and infants of various ethnicities [90, 106, 107]. Several polymorphisms within insulin growth factor 1 gene (*IGF1*), insulin growth factor 2 gene (*IGF2*) and the genes encoding their receptors have demonstrated a higher risk of PTB [68, 108, 109]. Both *IGF1* and *IGF2* encode growth factors similar in function to insulin, with roles in mediating growth, including fetoplacental development [110]. SNPs associated with sPTB within genes involved in glucose and protein homeostasis folate and methionine synthesis as well as the metabolism and storage of vitamin D and other minerals have been identified in PTB cases [109, 111–114]. A recent study of 254 Korean women also established the role of transcobalamin transport in PTB [115]. Genes involved in the detoxification and metabolism of organophosphates and xenobiotics have also shown an association with PTB [81, 116].

#### Hematological, Vascular, and Endothelial Pathway-Related Polymorphisms

Genes involved in producing coagulation factors have been investigated and are associated with PTB in some candidate gene studies [95, 117]. Genes that reduce thrombin formation, as well as both clot formation and degradation, have also been implicated in studies using infant samples [118, 119]. Prostaglandins have a well-established role in inducing myometrial contraction and are involved in inflammatory, angiogenic, and platelet aggregation pathways [120, 121]. A 2012 study assessed genes involved in prostaglandin synthesis and function including prostaglandin-endoperoxide synthase 1 (*PTGS1*), prostaglandin-endoperoxide synthase 2 (*PTGS2*), prostaglandin E synthase (*PTGES*), and prostaglandin E synthase 2 (*PTGES2*) genes [122]. The study included 542 preterm and 568 term African American mothers, adjusted for multiple lifestyle-related confounders, and identified several SNPs significantly associated with PTB. Similar findings have been established within *PTGS1*, *PTGS2*, prostaglandin E receptor 2 (*PTGER2*), and prostaglandin E receptor 3 (*PTGER3*) genes in maternal samples and infant samples [49, 69, 81, 123]. Polymorphisms within genes encoding growth factors involved in vasculogenesis, angiogenesis, endothelin, and endothelial growth have all been implicated in PTB [97, 124, 125]. Potential vascular smooth muscle relaxation via polymorphisms in nitric oxide synthase 2 gene (*NOS2*) and nitric oxide synthase 3 gene (*NOS3*) have also been associated with this phenotype [118, 126], supporting the role of the vascular and endothelial system in preterm delivery.

#### Limitations in Candidate Gene Studies

Candidate gene studies present a large body of research in PTB genetics and are relatively cost-effective with less sample size demands compared to other large-scale hypothesis-free studies such as GWAS. However, the findings are mostly inconclusive; studies commonly lacked adequate sample sizes and often did not conduct replication/validation analyses. Likewise, SNPs identified in candidate gene studies have not been replicated in large GWAS. Confounders were often not accounted for in these studies, despite knowledge that PTB is influenced by several environmental factors. Overall, published findings have been of minimal clinical utility thus far. Candidate gene studies are limited in that they are influenced by prior research and, due to study design, cannot identify novel variants involved in PTB. Online Resource 1 provides an overview of the genes identified in PTB-related candidate gene studies, grouped by biological pathway. A comprehensive catalogue of summary findings from the candidate gene studies is provided in Online Resource 2. Few mitochondrial SNPs have been studied; those associated with PTB are included in Online Resource 2.

### Genome-Wide Association Studies

A substantial improvement in understanding the genetics of complex diseases has occurred within recent decades with the implementation of GWAS. To our knowledge, 13 GWAS published in the last 10 years have assessed PTB phenotypes including sPTB, idiopathic PTB, PPROM, PTB at varying gestational ages, and gestational duration. Whilst there is a clinical overlap between PTB and decreased gestational duration, note that gestational duration also includes normal variations in birth timing and therefore the genetic variants identified in analyses of gestational duration may not play the same role in PTB.

A large maternal genome-wide meta-analysis has recently been conducted with sample sizes fivefold larger than previously published [127]. GWAS data from 18 cohorts was utilized, providing almost 200,000 European maternal samples for gestational duration analyses and over 270,000 samples for sPTB analyses (18,797 preterm and 260,245 term births). The study identified genetic variants at 22 loci that were associated with gestational duration and 6 loci that were associated with sPTB, at a genome-wide significance (defined as *P* <5×10^−08^ [128]). Whilst most identified loci were distributed between the two phenotypes, the study calculated the observed estimate of genetic correlation to be moderate (rg = −0.62, 95% confidence interval (CI) −0.72 to −0.51), indicating that there may be variations in the genetic effects on gestational duration versus sPTB. The authors also demonstrated that maternal genetics had a larger impact on PTB when compared to fetal genetics, although overall effect sizes were observed to be small. Subsequent analyses to evaluate the use of the variants identified to predict PTB phenotypes including GA and sPTB, expectedly, suggested poor predictive performances (area under the receiver operator characteristic (AUROC) = 0.61, OR = 0.69, 95% CI 0.56–0.85) [127].

Zhang et al. (2017) published a large GWAS that utilized DNA samples from over 40,000 European women (3331 preterm and 40,236 term births) from 23andMe and identified several genetic variants associated with sPTB or gestational duration in both discovery and replication analyses at a genome-wide significance. Polymorphisms located in EBF transcription factor 1 (*EBF1*), angiotensin II receptor type 2 (*AGTR2*), adenylate cyclase 5 (*ADCY5*), and ras-related protein rap-2c (*RAP2C*) genes were strongly associated with decreased gestational duration. SNPs within both *EBF1* and *AGTR2* were associated with an increased risk of sPTB (<37 weeks). Variants located within eukaryote elongation factor selenocysteine-tRNA-specific gene (*EEFSEC*) and Wnt family member 4 gene (*WNT4*) were associated with increased gestational duration, and deletion variations within SNPs in *EEFSEC* were also associated with a decreased risk of sPTB [129].

Recently, Gupta et al. (2022) published a GWAS that examined the relationship between the maternal genome and early PTB of various sub-phenotypes. The GWAS analyses of 310 Caucasian women compared sPTB, iPTB, and PPROM against both low-risk and high-risk term births, as determined by the absence or presence of a previous history of PTB, respectively. The study identified several polymorphisms associated with all three sub-phenotypes at a genome-wide significance [130].

To assess the fetal effects on timing of parturition, Lui et al. (2019) conducted a large fetal GWAS meta-analysis of gestational duration, early preterm (<34 weeks), preterm (<37 weeks), and post-term births. The study identified a polymorphism within cytoskeleton associated protein 2 like gene (*CKAP2L*), a gene involved in the pro-inflammatory pathway [131], that was protective against decreased gestational duration in combined discovery and replication analyses (*β* = 0.034, 95% CI 0.025–0.043, *P*=3.96×10^−14^). The GWAS also identified a SNP in LIM domain containing preferred translocation partner in lipoma gene (*LPP*) and a SNP in spermatogenesis associated 6 gene (*SPATA6*); both were associated with a 1.64-fold increased odds of early sPTB. However, the SNPs could not be replicated in an independent cohort due to insufficient power [131].

Infant data has also been analyzed in a GWAS that aimed to identify genetic polymorphisms associated with PTB <30 weeks’ gestation [132]. Within an African sub-cohort of 190 cases and 1684 controls, this study identified an intergenic SNP located between YWHAQ pseudogene 9 (*YWHAQP9*) and *RP11-136B18* that was associated with a 2.81-fold increased odds of PTB (*P*=4.5×10^−09^). The study also identified a second intergenic SNP located between family with sequence similarity 87 member A gene (*FAM87A*) and F-box protein 25 gene (*FBXO25*) that was associated with a 0.57-fold decreased odds of PTB within an American sub-cohort (*n*=1847) (*P*=3.71×10^−08^) [132].

An overview of the GWAS discussed in this review is provided in Table 3. Online Resource 3 includes further details of the GWAS published between 2011 and 2023 with genome-wide significant results. Several other PTB-related GWAS have been published that have not identified genetic variants associated with PTB at a genome-wide level, although some have published data that almost reach this *P*-value threshold [133–139]. It is likely that a subset of these studies did not identify genome-wide significant results due to the use of broadly and heterogeneously phenotyped cohorts that reflect different genetic pathways and relatively small sample sizes. Most GWAS discussed above analyzed iPTBs and pPROMs as a single group of sPTBs. Whilst these conditions share several etiological and clinical similarities, the events leading to both conditions are often distinct. Grouping the clinical conditions could be a limitation for GWAS and future studies should consider analyzing the sub-phenotypes separately. Another common limitation across published GWAS is the lack of successful replication with independent cohorts and among studies. Future studies with large sample sizes that utilize carefully phenotyped cohorts may increase the power needed to successfully identify significant loci with larger effect size. However, the economic and feasibility concerns associated with this pose challenges to conducting such studies; hence, few large-scale PTB-related GWAS have been conducted. The results from GWAS published to date have identified novel variants and biological pathways, advancing our understanding of the role of genetics in PTB. The challenge is translating these findings for clinical use to identify women at increased genetic risk of delivering preterm.

**Table 3:** Overview of genome-wide association studies (GWAS) published between 2011 and 2023 with genome-wide significant results (*P*<5×10^−08^)

Study	Genes identified	Sample	Total sample size	Ethnicities analyzed	Phenotypes analyzed
Solé-Navais, 2023 [127]	*ADCY5; AGTR2; COL27A1; EBF1; EEFSEC; FAF1; FBXO32; GDAP1; HAND2; HIVEP3; HLA-DQA1; KCNAB1; KDR; LEKR1; LRP5; LSM3; MRPS22; MYOCD; RAP2C; TCEA2; TET3; TFAP4; WNT4; ZBTB38*	Maternal	279043	European	sPTB <37 weeks, Gestational duration
Gupta, 2022 [130]	*LOC105377408; MAST1*	Maternal	310	European	Preterm prelabor rupture of membranes (PPROM) <34 weeks; spontaneous preterm birth (sPTB) <34 weeks
Huusko, 2021 [139]	*DNAJB8*	Maternal	43568	European	sPTB <37 weeks
Liu, 2019 [131]	*CKAP2L; IL1A; IL1B; NT5DC4*	Infant	84689	European	sPTB <34 weeks, Gestational duration
Rappoport, 2018 [132]	*YWHAQP9/RP11-136B18; FAM87A/FBXO25*	Infant	1847	African; American	sPTB <30wks
Zhang, 2017 [129]	*EBF1; EEFSEC; RUVBL1; TEKT3/PMP22/TVP23C-CDRT4; AGTR2/PLS3; WNT4; ADCY5; TET3; RAP2C/FRMD7/STK26*	Maternal	43567	European	sPTB <37 weeks; Gestational duration

### Whole-Exome Sequencing and Whole-Genome Sequencing Analyses

Recent advances in DNA sequencing technologies have allowed genetic association studies to conduct WES and WGS. However, to date, few WES or WGS studies have been performed to investigate the role of genetics in PTB, likely due to the increased costs of genetic sequencing [140].

Modi et al. (2017) performed whole-exome sequencing using neonatal DNA derived from PPROM cases (*n*=49) and term births (*n*=20) to identify candidate genes involved in extracellular matrix synthesis that may be associated with preterm labor. The subsequent candidate gene analyses conducted on 188 PPROM cases and 175 controls identified several risk variants in fibrillar collagen genes associated with PPROM [141]. Whilst the small sample size of the discovery phase is a limitation, the study presents an alternate approach to identify rare variants associated with PTB. Similar case-control studies have been published implicating rare variants in genes involved in the KEGG complement and coagulation cascade [86], as well as innate and host defense pathways [142]. Huusko et al. in 2018 conducted WES on families with predispositions to sPTB. Discovery analyses including samples from 17 Finnish women identified rare variants that significantly affected the glucocorticoid receptor signaling pathway. These findings were replicated in a validation cohort of 93 Danish sister pairs and two triads [143].

The small number of published WES/WGS studies using PTB samples has provided promising results, although most have been limited in sample size. As the cost of sequencing continues to decrease, the ability to conduct sequencing analyses using larger sample sizes may increase, potentially enabling the identification of multiple variants associated with this disease. Furthermore, sequencing data may allow better identification of functional variants rather than index variants that are only in linkage disequilibrium with the functional variants. The capability to detect rare and potentially functional variants in WES and WGS analyses may also enable future studies to identify polymorphisms with relatively large effect sizes that are associated with PTB.

### Copy Number Variant Analyses

CNVs involving genes that are sensitive to changes in copy numbers have the potential to cause genetic disorders, as previously established in diseases such as neuropsychiatric and neurodegenerative disorders, autism, and immune deficiency [144–147]. However, few studies have analyzed CNVs associated with PTB, with fewer obtaining significant results.

A 2017 study conducted WES and assessed transcript levels in 160 mothers with PTB (<35 weeks’ gestation) and identified several ubiquitin-proteasome-collagen (CUP) pathway-related mRNAs that were differentially expressed in cases. Whilst the study then identified four CNVs within CUP-related genes within sPTB samples, the CNV results were inconclusive, likely due to sample size constraints [148]. Uzun et al. (2016) conducted a genome-wide approach to analyze CNVs in 454 women who delivered preterm (<34 weeks’ gestation) and 1018 women who delivered at term. However, the study found no evidence of an increased burden of CNVs in preterm cases compared to those born at term [149]. In contrast, Zheng et al. (2013) analyzed 898 PTB cases and 978 term deliveries and identified CNVs associated with a significantly increased risk of PTB (<35 weeks’ gestation). The CNVs were located within glutathione s-transferase theta 2 (*GSTT2a*) and glutathione s-transferase theta 2b (*GSTT2b*) genes [150].

CNV analyses represent a small proportion of published PTB related genetic studies and further investigations should be conducted to establish the role of CNVs in PTB risk. Improvements in sequencing methods have enhanced precise detection of CNVs. However, clinical interpretation and determining the functional effects of CNVs poses an ongoing challenge for future studies [151].

### De Novo Mutation Analyses

There have been a limited number of studies that have investigated the role of fetal de novo mutations on PTB risk. A study published in 2017 conducted WGS analyses to assess fetal de novo mutations from 816 trio families. Data suggested that preterm infants (<37 weeks’ gestation) had a higher prevalence of de novo mutations than those born at term (*P*=6.9×10^−03^). The mutations were predominantly located in genes essential to embryonic development, especially fetal brain development [152]. A recent study conducted genome-wide genotyping and CNV calling on 488 infant cases and 3208 parent controls and identified 14 de novo CNV significantly associated with PTB (<32 weeks’ gestation). These CNV had a mutation rate of >2.9% compared to 2.1% in the control cohort (*P*=0.002) indicating that the mutations were significantly more common in those that experienced preterm delivery [153]. Both studies were underpowered and did not stratify analyses based on PTB sub-phenotypes. Nevertheless, they highlight new methods of extending PTB genetic risk and future analyses should consider examining rare fetal de novo mutations in larger, carefully phenotyped cohorts.

## Preterm Birth Transcriptomics

PTB-related transcriptomic studies have conducted analyses on a wide variety of tissues including the placenta, basal plate, myometrium, decidua, fetal membranes (chorion and amnion), cervix, umbilical cord, fetal blood, and maternal blood, as identified in a systematic review published in 2015 [154].

A study recently published by Gupta et al. (2022) performed genetic association and differential gene expression analyses using blood samples from pregnant women at 16- and 20-weeks gestation; the results from the GWAS are discussed in the PTB genetics section above. The study included 114 RNA samples for analyses and identified 147 differentially expression genes associated with PPROM in the samples taken at 20-week gestation, implicating genes involved in local inflammatory responses [130]. Studies have also identified associations between PTB and differentially expressed genes involved in both anti- and pro-inflammatory cytokine pathways [77, 155–158], the complement pathway [159, 160], and white blood cell function [161, 162]. An upregulation of cyclooxygenase 2 gene (*COX2*) has been identified in those with PTB [163, 164]; this results in increased prostaglandin production including prostaglandin F2α and prostaglandin E2, which have both been shown to induce cervical ripening and myometrial contractions [165]. Whilst the data supports the role of immune function and tissue remodeling in PTB, most studies have sampled women during preterm labor. Therefore, the data cannot be used to identify women earlier in pregnancy that are at risk of PTB.

Maternal peripheral blood sampled during pregnancy has been frequently utilized to conduct transcriptome profiling and develop risk prediction models. Zhou et al. in 2020 conducted a nested case-control study of 51 sPTB cases and 106 term births and utilized four *EBF1*-based microRNA (miRNA) in maternal blood sampled in the third-trimester to create a risk prediction score for sPTB with an AUROC of 0.82, sensitivity of 81%, and specificity of 72% [166]. *EBF1* gene expression and its association with PTB were further examined via transcript analyses that identified two *EBF1*-correlated lncRNA transcripts that were differentially expressed in sPTB [167]. A study published in 2017 utilized 30 miRNA from first-trimester peripheral blood mononuclear cells in a cohort of 39 pregnant women to create a risk score for developing sPTB. The study developed risk scores for early sPTB (<34 weeks’ gestation) and late sPTB (34 to 38 weeks’ gestation); the early sPTB risk score conferred an AUROC of 0.98 (*P*<0.0001) and AUROC for the late sPTB group of 0.92 (*P*<0.0001) [168]. Ngo et al. (2019) performed a similar study with 38 participants and highlighted seven RNA transcripts in maternal blood associated with PTB risk with an AUROC of 0.86 [169]. Studies have also developed similar risk scores using biological samples from multiple time points during pregnancy [170, 171] . These proof-of-concept studies provide promising results; however, the scores need to be validated in larger independent cohorts to confirm the predictive performance.

The performance of published transcriptomic predictive models using maternal blood samples is promising. Other studies have also successfully created predictive models utilizing RNA transcripts within tissues such as placenta, myometrium, and fetal membranes [172–174]. However, most studies have been limited by relatively small sample sizes, potentially resulting in inflated effect size estimations or false discovery rates. Nevertheless, there is a role in utilizing transcriptomic profiling in predicting PTB, and it is likely that risk prediction models may improve through the incorporation of clinical data.

## Preterm Birth Epigenetics

A limited number of epigenetic studies have been conducted utilizing maternal samples. In a cohort of 300 African American women, DNA methylation changes in maternal blood were significantly associated with early sPTB within genes involved in the immune response and cell differentiation processes [175]. Ross et al. (2020) examined the relationship between advanced biological age (measured using epigenetic aging indices) and gestational duration. The study utilized whole blood samples from 77 women and found that higher levels of the epigenetic indices in pregnancy were associated with shorter gestational length [176].

The epigenetic changes associated with PTB development have also been examined utilizing newborn blood samples. Merid et al. (2020) conducted epigenome-wide meta-analyses utilizing cord blood from 3648 newborns in 17 cohorts. The study identified 8999 CpG sites, annotated to 4966 genes, associated with gestational age. The annotated genes were broadly involved in processes critical for embryonic development [177]. A recent study conducted epigenome-wide analyses in two independent cohorts (combined *n*=502) to assess DNA methylation in cord blood that was significantly associated with gestational age of birth. Differentially methylated regions were identified and mapped to genes involved in the inflammatory and innate immune systems [178]. Other studies have also identified differential methylation regions and CpG sites in genes involved in the inflammatory and immune systems [179, 180], as well as embryonic development, metabolic processes, and parturition [181–184].

Whilst the infant studies have identified several epigenetic modifications that may be associated with PTB, there is a significant lack of epigenetic studies utilizing maternal samples. Of those published, the findings are somewhat inconclusive and provide little use in clinical settings. Studies of larger sample size may be more successful in identifying epigenetic biomarkers associated with PTB.

## Multiple “-Omics” Analyses

The majority of PTB-related “-omics” studies have analyzed a single set of “-omics” data such as genomics, transcriptomics, and epigenomics, as discussed in this review. Other “-omics” studies have also been performed in PTB-related studies but have not been reviewed in this paper. Systematic reviews of PTB biomarkers using multiplex, proteomic, metabolomic, and microbiomic approaches have been published [51, 185–187]. Common limitations across the single “-omics” studies are the lack of replication and that currently the findings have not been transferrable to clinical settings. Recently, some studies have integrated multiple “-omics” datasets to analyze the complex phenotype of PTB.

Huusko et al. in 2021 analyzed large genetic and transcriptomic datasets from mothers, infants, and placentas to examine the role of heat shock proteins and nuclear hormone receptors in sPTB. The study investigated 146 genes related to these proteins and receptors due to their potential involvement in stress responses such as immune activation. Several genes were associated with sPTB at a genome-wide significance. Transcriptomic results identified that the expression of these genes change in PTB and suggested that the activation of the specific heat shock proteins may be implicated with increased risk of prematurity [139].

A 2019 study utilized whole blood samples from 270 PTB case and 521 controls to integrate findings from WGS, RNA sequencing, and DNA methylation data [188]. The data were integrated and analyzed, and the results identified 160 significant genomic variants associated with various PTB-related phenotypes at a genome-wide significance. The identified genes were involved in biological pathways related to inflammation and immune function, and chemokine, interferon gamma, notch-1, endothelial growth factor receptor, and prolactin signaling [188].

Jehan et al. in 2020 conducted transcriptomic and proteomic profiling in plasma and metabolic profiling from urine samples in 81 pregnant women. Transcriptomic, proteomic, and metabolomic analyses predicted PTB with similar predictive performance (AUROC = 0.73, 0.75, and 0.59, respectively) [189]. However, an integrated model of all three datasets improved the results with an AUROC of 0.83. This potentially suggests that PTB is influenced by processes and biological systems at several levels that probably operate somewhat independently, and that combining datasets increases risk prediction performance [189]. However, Tarca et al. (2021) developed similar predictive models using transcriptomic and proteomic sampling and found that combining the separate predictive models did not change predictive performance [190].

The current body of multiple “-omics” research in the field of PTB is limited and the data from published studies were often not replicated in independent cohorts. This is potentially due to the computational challenges of conducting integrated analyses as well as the logistical concerns in obtaining data from multiple biological samples of the same pregnancies. Heterogeneity in the definition of the phenotype, biological samples assayed and time of collection, assay approaches and instrumentation, and analytical strategies can complicate integrated data analysis when studies do not follow a standard criterion or follow specific guidelines. Continuing advances in artificial intelligence coupled with well-planned study designs, model development, appropriate feature selection and extraction, and increasing sample size certainly has the potential to allow the development of improved predictive models for PTB. However, limitations exist with the lack of a suitable study cohort or a suitable database, and the ability to afford the computational demands needed to perform the analyses [191]. Overall, the results from the multiple “-omics” studies are promising and show potential to overcome current challenges in identifying clinical tools to predict PTB provided the limitations of these analyses are addressed in future studies.

## The Future of PTB Genetics Research

The existing body of research supports a possible role of inflammatory, immunological, metabolic, endocrine, tissue remodeling, vascular, and endothelial pathways in PTB, providing some insight into the pathophysiological processes underpinning this disease. Transcriptomic and epigenetic studies have been moderately successful in identifying PTB biomarkers of interest. Large GWAS have identified several replicated polymorphisms associated with PTB, and the biological significance of these findings via laboratory tissue analyses should be further investigated. However, the clinical translational value of published findings so far has been minimal. Most published studies have been limited in power and have often lacked replication in independent cohorts. Multiple studies have not adjusted for confounders or multiple testing, decreasing the validity of results. Heterogenous ethnic groups were also commonly used despite knowledge that ethnicity influences PTB risk. Moreover, studies that utilized mixed ethnic groups often did not adjust appropriately. Numerous studies also grouped PTB phenotypes into one large cohort, rather than disaggregate based on sub-phenotypes. Given the complexity of PTB, this may have heterogenized study cohorts, leading to decreased ability to detect true associations.

Current approaches are yet to work due to phenotyping, ethnic, and analytic issues, as well as limitations in sample size. Combining study cohorts, likely through the use of public databases or international collaborations, may mitigate some limitations with sample size requirements. However, this poses a feasibility challenge as currently, aside from GWAS, there is minimal use of catalogues and public databases. Many studies, particularly candidate gene studies, have also not provided the necessary information to utilize their results, such as direction of effect and risk alleles. Regardless of the feasibility challenges, the research community should consider sample size and phenotyping requirements when designing future studies. Our review has also highlighted the promising performance of multiple “-omics” analyses in creating effective prediction tools for PTB. It is likely that integrating multiple “-omics” datasets is a suitable approach to overcome the limitations in analyzing the complex disease of PTB. Integrating genetic data with clinical data, such as ultrasound findings and other clinical biomarkers, and environmental risk factors, may also yield tools with higher predictive performance given the multifactorial nature of PTB. Overall, future research needs to be conducted using large cohorts that are carefully phenotyped and should consider integrating multiple biological datasets.

## Conclusion

PTB is a global obstetric concern, affecting almost 15 million babies globally every year. The development of PTB is multifactorial with influence from both environmental and genetic factors. There is a large body of research exploring PTB genetics, with a particular focus on identifying associated genetic polymorphisms. A smaller subset of studies has investigated transcriptomic and epigenetic involvement in PTB. This review has summarized the body of literature regarding the role of genetics in PTB and has highlighted the need for conducting future research with large sample sizes, detailed phenotyping, and integrated multi “-omics” analyses with clinical biomarkers. Adopting these approaches may enable the successful development of prediction tools for PTB. This is critical to ensure that clinicians can determine individualized PTB risk and stratify clinical care to prevent PTB.

### Supplementary Information

ESM 1

ESM 2

ESM 3
