# Supplementary material for: The Role of Genetics in Preterm Birth
Source: Reprod Sci. 2023 Jul 14;30(12):3410–27. doi: 10.1007/s43032-023-01287-9 (PMC10692032; doi:10.1007/s43032-023-01287-9)
Supplement: Supplementary file 1 — ESM 1 [file 43032_2023_1287_MOESM1_ESM.pdf]

Manuscript Title: The role of genetics in preterm birth

Journal: Reproductive Sciences

Authors: Elyse Mead, Carol Wang, Jason Phung, Joanna YX Fu, Scott Williams, Mario Merialdi, Bo Jacobsson, Stephen Lye, Ramkumar Menon, Craig Pennell*1,2,3

*Corresponding author email address: craig.pennell@newcastle.edu.au

[1] School of Medicine and Public Health, University of Newcastle, Newcastle, NSW, 2308, Australia,

[2] Hunter Medical Research Institute, Newcastle, NSW, 2305, Australia,

[3] Department of Maternity and Gynaecology, John Hunter Hospital, Newcastle, NSW, 2305 Australia

Online Resource 1: Candidate genes grouped by biological pathway

The table is grouped based on biological pathway and ordered alphabetically based on gene symbol. The table includes references of case-control or cohort candidate gene studies (reference list at end of document) that identified significant polymorphisms within the listed genes that were associated with PTB using maternal samples only, infant samples only, or both maternal and infant samples. The list of genes was extracted from Online Resource 2 (ESM_2.xlsx). Genes are listed as gene symbol and grouped by pathway based on summary of gene function as per (Stelzer et al. 2016). Genes that could not be grouped into one of the first four pathways were allocated into the "Miscellaneous" group.

Pathway	*Gene*	Maternal only	Infant only	Both
Immunological and Inflammatory	*ALOX5*	(Liu et al. 2012)		
	ALOX5AP	(Liu et al. 2012)		
	CARD15	(Ryckman et al. 2010)		
	CCL2	(Velez et al. 2008a)		
	CCR2		(Romero et al. 2010a)	
	CD55		(Romero et al. 2010a)	
	C4BPA	(McElroy et al. 2013)		
	CR1	(McElroy et al. 2013)		
	CSF1	(Romero et al. 2010a)		
	CTLA4	(Velez et al. 2008a)		
	DEFB1	(Romero et al. 2010a)		
	FAS	(Fuks et al. 2005)		
	FcγRIIb	(Iwanaga et al. 2011)		
	HLA-DQA1	(Falah et al. 2013)		
	HLA-DRA	(Falah et al. 2013)		
	HLA-DRB1	(Falah et al. 2013)		
	ICAM-1	(Kwon et al. 2009)		
	IFNγ			(Speer et al. 2006)
	IFNGR1		(Romero et al. 2010b)	
	IFNGR2	(Harmon et al. 2013)		
	IL1α	(Sata et al. 2008)	(Velez et al. 2008a)	
	IL1β	(Hollegaard et al. 2008; Belousova et al. 2019; Pereyra et al. 2016)	(Yılmaz et al. 2012) (Velez et al. 2008a; Genç et al. 2002)	
	IL1R1	(Velez et al. 2008a)		

	IL1R2	(Langmia et al. 2015b) (Velez et al. 2008a)		
	IL1RAP			(Velez et al. 2008a)
	IL1RN	(Gillespie et al. 2017) (Velez et al. 2008a)		
	IL2		(Romero et al. 2010b)	
	IL2RA	(Velez et al. 2008a)		
	IL2RB		(Velez et al. 2008a; Velez et al. 2009)	
	IL4	(Harmon et al. 2013; Annells et al. 2004)	(Velez et al. 2008a)	
	IL4R			(Velez et al. 2008a)
	IL5			(Velez et al. 2008a)
	IL6	(Karakaş et al. 2018; Gómez et al. 2010; Pereyra et al. 2016; Simhan et al. 2003)	(Fortunato et al. 2008)	
	IL6R	(Velez et al. 2008b; Velez et al. 2007; Fortunato et al. 2008; Velez et al. 2008a)	(Romero et al. 2010b)	
	IL8RA	(Ryckman et al. 2010)		
	IL10	(Han et al. 2020; Lyubomirskaya et al. 2020a; Lyubomirskaya et al. 2020b; Pandey et al. 2018; Annells et al. 2004; Velez et al. 2008a)		(Ramos et al. 2016)
	IL10RA			(Velez et al. 2008a)
	IL10RB		(Ryckman et al. 2010)	
	IL12α	(Harmon et al. 2013)		
	IL13	(Harmon et al. 2013)	(Heinzmann et al. 2009)	
	IL15	(Velez et al. 2009)		
	IL16	(Frey et al. 2016)		
	IL18	(Velez et al. 2008a)	(Krueger et al. 2011)	
	IL18BP		(Romero et al. 2010a)	
	IL23R	(Falah et al. 2013)		
	KIR3DL2	(Harmon et al. 2013)		
	LTA	(Engel et al. 2005)		
	LTF	(Romero et al. 2010b)		
	MBL2	(Silva et al. 2020; Annells et al. 2004)	(Bodamer et al. 2006)	
	MCP1		(Wang et al. 2015)	
	MICA		(Song et al. 2017)	
	NFKB1			(Ryckman et al. 2010)
	NFKBIA			(Velez et al. 2008a)
	NFKBIB			(Velez et al. 2008a)
	NFKBIE	(Ryckman et al. 2010)		
	NOS2A		(Gibson et al. 2007)	
	PLAT			(Velez et al. 2008a)
	PLA2G4A	(Velez et al. 2008a)		
	PTGES	(Liu et al. 2012)		
	PTGES2	(Liu et al. 2012)		
	SFTPD		(Karjalainen et al. 2012a)	
	TIRAP	(Karody et al. 2013)		

	TLR1	(Romero et al. 2010a)	(Romero et al. 2010b)	
	TLR2	(Ramos et al. 2016; Velez et al. 2008a; Romero et al. 2010b)		
	TLR4		(Bitner et al. 2013; Lorenz et al. 2002)	
	TLR7	(Velez et al. 2008a)		
	TNFα	(As Sayaril et al. 2018; Drews-Piasecka et al. 2014; Gebhardt et al. 2009; Harmon et al. 2013; Harper et al. 2011; Hollegaard et al. 2008; Amory et al. 2004; Macones et al. 2004; Roberts et al. 1999)	(Aidoo et al. 2001; Chen et al. 2003; Fortunato et al. 2008; Menon et al. 2006b)	(Yılmaz et al. 2012)
	TNFR1	(Menon et al. 2006b; Menon et al. 2006a)		(Fortunato et al. 2008)
	TNFR2	(Jones et al. 2012; Fortunato et al. 2008; Menon et al. 2006b; Menon et al. 2006a)		
	TREM1		(Velez et al. 2008a)	
Tissue remodelling	*AR*		(Karjalainen et al. 2012b)	
	B2AR	(Doh et al. 2004)		
	CGA	(Plunkett et al. 2011)		
	COL1A1	(Velez et al. 2008a)		
	COL1A2	(Ryckman et al. 2010)		(Velez et al. 2008a)
	COL3A1			(Velez et al. 2008a)
	COL4A1		(Romero et al. 2010b)	
	COL4A2			(Romero et al. 2010b)
	COL4A3		(Gimenez et al. 2017)	
	COL4A4			(Romero et al. 2010b)
	COL4A5		(Romero et al. 2010b)	
	COL5A1	(Velez et al. 2008a)		
	COL5A2			(Velez et al. 2008a)
	CRH		(Ryckman et al. 2010)	
	CRHBP			(Velez et al. 2008a)
	CRHR2	(Schmid et al. 2010)	(Velez et al. 2008a)	
	CSPG2	(Romero et al. 2010a)		
	FGF1		(Preda et al. 2020)	(Romero et al. 2010b)
	FGF4	(Romero et al. 2010a)		
	FN1		(Romero et al. 2010b)	
	FSHR	(Chun et al. 2013; Plunkett et al. 2011)	(Dominguez-Lopez et al. 2018)	
	IGF2		(Romero et al. 2010b)	
	MMP1		(Wang et al. 2008)	(Velez et al. 2008a)
	MMP3			(Velez et al. 2008a)
	MMP8		(Velez et al. 2008a)	
	MMP9	(Jones et al. 2012; Pandey and Awasthi 2020)		
	MMP10	(Romero et al. 2010a; Romero et al. 2010b)		
	MMP16	(Romero et al. 2010b)		
	MMP19		(Romero et al. 2010a)	

	PGR	(Langmia et al. 2015c; Manuck et al. 2010)	(Velez et al. 2008a)	(Kadivnik et al. 2022)
	PGRMC1	(Velez et al. 2008a)		
	RLN2	(Lyubomirskaya et al. 2020a; Lyubomirskaya et al. 2020b; Rocha et al. 2013; Vogel et al. 2009)		
	SERPINH1		(Wang et al. 2006)	
	TIMP1		(Romero et al. 2010b)	
	TIMP2	(Romero et al. 2010a; Frey et al. 2016)		(Romero et al. 2010b)
	TIMP3			(Velez et al. 2008a)
	TIMP4		(Ryckman et al. 2010)	
	TNR	(Romero et al. 2010a)	(Romero et al. 2010b)	
Metabolic	*ADH1B*			(Ryckman et al. 2010)
	CETP		(Romero et al. 2010b)	
	CRHR1		(Gimenez et al. 2017)	
	CYP19A1			(Velez et al. 2008a)
	CYP2D6			(Velez et al. 2008a)
	DHFR	(Velez et al. 2008a)		
	EPHX1	(Ryckman et al. 2010)		
	FADS2	(Liu et al. 2012)		
	GC	(Wang et al. 2021)		
	GSTP1		(Velez et al. 2008a)	
	HSD11B1	(Velez et al. 2008a)		
	HSD17B7		(Ryckman et al. 2010)	
	IGF1	(He et al. 2017)	(Velez et al. 2008a)	
	IGF1R		(Haataja et al. 2011)	
	IRS1		(Romero et al. 2010b)	
	LEP			(Salem et al. 2016)
	LPL	(Falah et al. 2013)		
	LRP2	(Wang et al. 2021)		
	MTHFR	(Hwang et al. 2017; Nan and Li 2015; Tiwari et al. 2015; Valdez et al. 2004)	(Zhu et al. 2015)	
	MTRR	(Engel et al. 2006)		
	NAT1	(Velez et al. 2008a)		
	PLA2G4D	(Liu et al. 2017)		
	PON1		(Gimenez et al. 2017; Ryckman et al. 2010; Velez et al. 2008a)	
	PPARG	(Meirhaeghe et al. 2007)		
	SHMT1	(Engel et al. 2006)		
	SLC23A1		(Velez et al. 2008a)	
	SLC23A2	(Erichsen et al. 2006)		
	TCN2	(Kwon et al. 2021)		
	TSHR			(Velez et al. 2008a)
	VDR	(Barchitta et al. 2018; Javorski et al. 2018; Rosenfeld et al. 2017; Manzon et al. 2014)		(Dutra et al. 2020)
Haematological, vascular, and endothelial	*ACE*	(Romero et al. 2010a)		
	AGT	(Romero et al. 2010b)		
	AGTR			(Romero et al. 2010b)
	ANG	(Romero et al. 2010a)		
	EDN2		(Velez et al. 2008a)	

	ERG	(Plunkett et al. 2011)		
	F2		(Chen et al. 2007)	
	F2R		(Ryckman et al. 2010)	
	F3		(Gimenez et al. 2017)	
	F5	(Yu et al. 2009; McElroy et al. 2013; Velez et al. 2008a)		
	F7	(Velez et al. 2008a)		
	F10	(Ryckman et al. 2010)		
	FLT1	(Frey et al. 2016)		
	MHC	(Falah et al. 2013)		
	NOS3	(Silva et al. 2020)	(Velez et al. 2008a)	
	NPPA		(Romero et al. 2010b)	
	NPR1		(Romero et al. 2010b)	
	PAR1	(Grisaru-Granovsky et al. 2007)		
	PLAUR		(Ryckman et al. 2010)	
	PLG	(Ryckman et al. 2010)		
	PTGER1	(Romero et al. 2010a)		
	PTGER3	(Ryckman et al. 2010)		(Velez et al. 2008a)
	PTGFR			(Ryckman et al. 2010)
	PTGS1	(Liu et al. 2012; Velez et al. 2008a)		
	PTGS2	(Liu et al. 2012)	(Velez et al. 2008a)	
	REN	(Romero et al. 2010a)		(Romero et al. 2010b)
	SELE	(Romero et al. 2010b)		
	SERPINC1		(Romero et al. 2010b)	
	SERPINE1		(Chen et al. 2007)	
	TBXAS1	(Romero et al. 2010b)		
	TFPI		(Ryckman et al. 2010)	
	THBD		(Gibson et al. 2007)	
	THPO	(Romero et al. 2010b)		
	VEGFA	(Langmia et al. 2015a; Velez et al. 2008a)		
	VEGFC	(Romero et al. 2010b)		
	VWF	(Romero et al. 2010a)		
Miscellaneous	*ADRB2*		(Gibson et al. 2007)	
	AKAP10	(Langmia et al. 2015d)		
	AP3M2			(Velez et al. 2008a)
	CBS		(Velez et al. 2008a)	
	CCHCR1	(Falah et al. 2013)		
	CDKN2	(Falah et al. 2013)		
	CENTG2	(Plunkett et al. 2011)		
	COMT		(Thota et al. 2012)	
	CPNE4	(Plunkett et al. 2011)		
	DUSP16	(Plunkett et al. 2011)		
	ELN		(Romero et al. 2010b)	
	EMR1	(Plunkett et al. 2011)		
	EPHX2			(Velez et al. 2008a)
	FHIT	(Plunkett et al. 2011)		
	GABRG3	(Plunkett et al. 2011)		
	GNB3	(Romero et al. 2010b)		
	HSPA14		(Velez et al. 2008a)	
	HSPA1B		(Ryckman et al. 2010)	
	HSPA6		(Velez et al. 2008a)	
	HSPG2	(Romero et al. 2010b)		

	HTR2A	(Romero et al. 2010b)		
	IGF2R		(Romero et al. 2010b)	
	IMP5		(Romero et al. 2010a)	
	KCNN3	(Day et al. 2011)	(Gimenez et al. 2017)	
	KCNN4	(Day et al. 2011)		
	KL			(Velez et al. 2008a)
	LDB2	(Plunkett et al. 2011)		
	LMO1	(Plunkett et al. 2011)		
	LPA		(Romero et al. 2010b)	
	MTHFD1	(Velez et al. 2008a)		
	NPAS3	(Plunkett et al. 2011)		
	NR3C1		(Velez et al. 2008a)	
	NR3C2	(Christiaens et al. 2015)		
	PAFAH1B1		(Velez et al. 2008a)	
	PAX9	(Plunkett et al. 2011)		
	PER3	(Kovac et al. 2019)		
	PGEA1		(Ryckman et al. 2010)	
	PGRMC2	(Ryckman et al. 2010)		
	PLA2G4C	(Plunkett et al. 2010)		
	PON2			(Velez et al. 2008a)
	PRKCA	(Frey et al. 2016; Gómez et al. 2010)		
	PTPRD	(Plunkett et al. 2011)		
	RASD2	(Plunkett et al. 2011)		
	RHOBTB3	(Plunkett et al. 2010)		
	SCNN1A	(Velez et al. 2008a)		
	SLC6A4			(Ryckman et al. 2010)
	SEPS1		(Wang et al. 2013)	
	SHP2		(Shim et al. 2017)	
	SKA2	(Ijabi et al. 2019)		
	SLC35B2		(Velez et al. 2008a)	
	TERT	(Marrs et al. 2018)		
	TEX12	(Velez et al. 2008a)		
	TGFB1	(Speer et al. 2006)		
	THBS4	(Romero et al. 2010b)		
	UGT1A1			(Velez et al. 2008a)
	VRK2	(Plunkett et al. 2011)		
	WWOX	(Plunkett et al. 2011)		

REFERENCES

Aidoo M, McElroy PD, Kolczak MS, Terlouw DJ, Ter Kuile FO, Nahlen B, Lal AA, and Udhayakumar V. Tumor necrosis factor-? promoter variant 2 (TNF2) is associated with pre-term delivery, infant mortality, and malaria morbidity in western Kenya: Asembo Bay Cohort Project IX. *Genetic Epidemiology* 2001: **21**; 201-211.

Amory JH, Adams KM, Lin MT, Hansen JA, Eschenbach DA, and Hitti J. Adverse outcomes after preterm labor are associated with tumor necrosis factor-alpha polymorphism -863, but not -308, in mother-infant pairs. *Am J Obstet Gynecol* 2004: **191**; 1362-1367.

Annells MF, Hart PH, Mullighan CG, Heatley SL, Robinson JS, Bardy P, and McDonald HM. Interleukins-1, -4, -6, -10, tumor necrosis factor, transforming growth factor-beta, FAS, and mannose-binding protein C gene polymorphisms in Australian women: Risk of preterm birth. *Am J Obstet Gynecol* 2004: **191**; 2056-2067.

As Sayaril N, Bhat RS, Warsy AS, Babay ZA, Addar MH, Shaik J, Al-Marry B, and Al-Daihan S. Polymorphisms in proinflammatory cytokine genes, effect on gene expression and association with preterm delivery in Saudi females. *Cell Mol Biol (Noisy-le-grand)* 2018: **64**; 55-60.

Barchitta M, Maugeri A, La Rosa M, Magnano San Lio R, Favara G, Panella M, Cianci A, and Agodi A. Single Nucleotide Polymorphisms in Vitamin D Receptor Gene Affect Birth Weight and the Risk of Preterm Birth: Results From the "Mamma & Bambino" Cohort and A Meta-Analysis. *Nutrients* 2018: **10**; 1172.

Belousova VS, Svitich OA, Timokhina EV, Strizhakov AN, and Bogomazova IM. Polymorphism of the IL-1β, TNF, IL-1RA and IL-4 Cytokine Genes Significantly Increases the Risk of Preterm Birth. *Biochemistry (Mosc)* 2019: **84**; 1040-1046.

Bitner A, Sobala W, and Kalinka J. Association Between Maternal and Fetal TLR4 (896A>G, 1196C>T) Gene Polymorphisms and the Risk of Pre-term Birth in the Polish Population. *American Journal of Reproductive Immunology* 2013: **69**; 272-280.

Bodamer OA, Mitterer G, Maurer W, Pollak A, Mueller MW, and Schmidt WM. Evidence for an association between mannose-binding lectin 2 (MBL2) gene polymorphisms and pre-term birth. *Genetics in Medicine* 2006: **8**; 518-524.

Chen BH, Carmichael SL, Shaw GM, Iovannisci DM, and Lammer EJ. Association between 49 infant gene polymorphisms and preterm delivery. *American Journal of Medical Genetics Part A* 2007: **143A**; 1990-1996.

Chen D, Hu Y, Wu B, Chen L, Fang Z, Yang F, and Wang L. Tumor necrosis factor-alpha gene G308A polymorphism is associated with the risk of preterm delivery. *Beijing Da Xue Xue Bao Yi Xue Ban* 2003: **35**; 377-381.

Christiaens I, Ang QW, Gordon LN, Fang X, Williams SM, Pennell CE, and Olson DM. Two novel genetic variants in the mineralocorticoid receptor gene associated with spontaneous preterm birth. *BMC Medical Genetics* 2015: **16**.

Chun S, Plunkett J, Teramo K, Muglia LJ, and Fay JC. Fine-Mapping an Association of FSHR with Preterm Birth in a Finnish Population. *PLoS ONE* 2013: **8**; e78032.

Day LJ, Schaa KL, Ryckman KK, Cooper M, Dagle JM, Fong C-T, Simhan HN, Merrill DC, Marazita ML, Murray JC, *et al.* Single-Nucleotide Polymorphisms in the KCNN3 Gene Associate With Preterm Birth. *Reproductive Sciences* 2011: **18**; 286-295.

Doh K, Sziller I, Vardhana S, Kovacs E, Papp Z, and Witkin SS. Beta2-adrenergic receptor gene polymorphisms and pregnancy outcome. *J Perinat Med* 2004: **32**; 413-417.

Dominguez-Lopez P, Diaz-Cueto L, Arechavaleta-Velasco M, Caldiño-Soto F, Ulloa-Aguirre A, and Arechavaleta-Velasco F. The follicle-stimulating hormone receptor Asn680Ser polymorphism is associated with preterm birth in Hispanic women. *The Journal of Maternal-Fetal & Neonatal Medicine* 2018: **31**; 580-585.

Drews-Piasecka E, Seremak-Mrozikiewicz A, Barlik M, Kurzawińska G, Wolski H, Woyciechowska A, Czerny B, and Drews K. The significance of TNF-alpha gene polymorphisms in preterm delivery. *Ginekol Pol* 2014: **85**; 428-434.

Dutra LV, Affonso-Kaufman FA, Cafeo FR, Kassai MS, Barbosa CP, Santos Figueiredo FW, Suano-Souza FI, and Bianco B. Association between vitamin D plasma concentrations and VDR gene variants and the risk of premature birth. *BMC Pregnancy and Childbirth* 2020: **20**.

Engel SA, Erichsen HC, Savitz DA, Thorp J, Chanock SJ, and Olshan AF. Risk of spontaneous preterm birth is associated with common proinflammatory cytokine polymorphisms. *Epidemiology* 2005: **16**; 469-477.

Engel SM, Olshan AF, Siega-Riz AM, Savitz DA, and Chanock SJ. Polymorphisms in folate metabolizing genes and risk for spontaneous preterm and small-for-gestational age birth. *American Journal of Obstetrics and Gynecology* 2006: **195**; 1231.e1231-1231.e1231.

Erichsen HC, Engel SAM, Eck PK, Welch R, Yeager M, Levine M, Siega-Riz AM, Olshan AF, and Chanock SJ. Genetic Variation in the Sodium-dependent Vitamin C Transporters, SLC23A1, and SLC23A2 and Risk for Preterm Delivery. *American Journal of Epidemiology* 2006: **163**; 245-254.

Falah N, McElroy J, Snegovskikh V, Lockwood CJ, Norwitz E, Murray JC, Kuczynski E, Menon R, Teramo K, Muglia LJ*, et al.* Investigation of genetic risk factors for chronic adult diseases for association with preterm birth. *Human Genetics* 2013: **132**; 57-67.

Fortunato SJ, Menon R, Velez DR, Thorsen P, and Williams SM. Racial disparity in maternal-fetal genetic epistasis in spontaneous preterm birth. *Am J Obstet Gynecol* 2008: **198**; 666.e661-669; discussion 666.e669-610.

Frey HA, Stout MJ, Pearson LN, Tuuli MG, Cahill AG, Strauss JF, Gomez LM, Parry S, Allsworth JE, and Macones GA. Genetic variation associated with preterm birth in African-American women. *American Journal of Obstetrics and Gynecology* 2016: **215**; 235.e231-235.e238.

Fuks A, Parton LA, Polavarapu S, Netta D, Strassberg S, Godi I, and Hsu CD. Polymorphism of Fas and Fas ligand in preterm premature rupture of membranes in singleton pregnancies. *Am J Obstet Gynecol* 2005: **193**; 1132-1136.

Gebhardt S, Bruiners N, and Hillermann R. A novel exonic variant (221delT) in the LGALS13 gene encoding placental protein 13 (PP13) is associated with preterm labour in a low risk population. *Journal of Reproductive Immunology* 2009: **82**; 166-173.

Genç MR, Gerber S, Nesin M, and Witkin SS. Polymorphism in the interleukin-1 gene complex and spontaneous preterm delivery. *American Journal of Obstetrics and Gynecology* 2002: **187**; 157-163.

Gibson CS, MacLennan AH, Dekker GA, Goldwater PN, Dambrosia JM, Munroe DJ, Tsang S, Stewart C, and Nelson KB. Genetic polymorphisms and spontaneous preterm birth. *Obstet Gynecol* 2007: **109**; 384-391.

Gillespie SL, Neal JL, Christian LM, Szalacha LA, McCarthy DO, and Salsberry PJ. Interleukin-1 Receptor Antagonist Polymorphism and Birth Timing. *Nursing Research* 2017: **66**; 95-104.

Gimenez LG, Momany AM, Poletta FA, Krupitzki HB, Gili JA, Busch TD, Saleme C, Cosentino VR, Pawluk MS, Campaña H*, et al.* Association of candidate gene polymorphisms with clinical subtypes of preterm birth in a Latin American population. *Pediatric Research* 2017: **82**; 554-559.

Gómez LM, Sammel MD, Appleby DH, Elovitz MA, Baldwin DA, Jeffcoat MK, Macones GA, and Parry S. Evidence of a gene-environment interaction that predisposes to spontaneous preterm birth: a role for asymptomatic bacterial vaginosis and DNA variants in genes that control the inflammatory response. *Am J Obstet Gynecol* 2010: **202**; 386.e381-386.

Grisaru-Granovsky S, Tevet A, Bar-Shavit R, Salah Z, Elstein D, Samueloff A, and Altarescu G. Association study of protease activated receptor 1 gene polymorphisms and adverse pregnancy outcomes: Results of a pilot study in Israel. *American Journal of Medical Genetics Part A* 2007: **143A**; 2557-2563.

Haataja R, Karjalainen MK, Luukkonen A, Teramo K, Puttonen H, Ojaniemi M, Varilo T, Chaudhari BP, Plunkett J, Murray JC*, et al.* Mapping a New Spontaneous Preterm Birth Susceptibility Gene, IGF1R, Using Linkage, Haplotype Sharing, and Association Analysis. *PLoS Genetics* 2011: **7**; e1001293.

Han SH, Lee NR, Kim HJ, Kang YD, Kim JS, Park JW, and Jin HJ. Association between the IL-6, IL-10, and TNFα gene polymorphisms and preterm-birth in Korean women. *Genes & Genomics* 2020: **42**; 743-750.

Harmon QE, Engel SM, Olshan AF, Moran T, Stuebe AM, Luo J, Wu MC, and Avery CL. Association of Polymorphisms in Natural Killer Cell-Related Genes With Preterm Birth. *American Journal of Epidemiology* 2013: **178**; 1208-1218.

Harper M, Zheng SL, Thom E, Klebanoff MA, Thorp J, Sorokin Y, Varner MW, Iams JD, Dinsmoor M, Mercer BM, *et al.* Cytokine Gene Polymorphisms and Length of Gestation. *Obstetrics & Gynecology* 2011: **117**; 125-130.

He J-R, Lai Y-M, Liu H-H, Liu G-J, Li W-D, Fan X-J, Wei X-L, Xia X-Y, Kuang Y-S, Liu X-D, *et al.* Maternal IGF1 and IGF1R polymorphisms and the risk of spontaneous preterm birth. *Journal of Clinical Laboratory Analysis* 2017: **31**; e22125.

Heinzmann A, Mailaparambil B, Mingirulli N, and Krueger M. Association of interleukin-13/-4 and toll-like receptor 10 with preterm births. *Neonatology* 2009: **96**; 175-181.

Hollegaard MV, Grove J, Thorsen P, Wang X, Mandrup S, Christiansen M, Norgaard-Pedersen B, Wojdemann KR, Tabor A, Attermann J, *et al.* Polymorphisms in the tumor necrosis factor alpha and interleukin 1-beta promoters with possible gene regulatory functions increase the risk of preterm birth. *Acta Obstetricia et Gynecologica Scandinavica* 2008: **87**; 1285-1290.

Hwang IW, Kang YD, Kwon BN, Hong JH, Han SH, Kim JS, Park JW, and Jin HJ. Genetic variations of MTHFR gene and their association with preterm birth in Korean women. *Medicina* 2017: **53**; 380-385.

Ijabi J, Moradi-Sardareh H, Afrisham R, Seifi F, and Ijabi R. SKA2 gene - A novel biomarker for latent anxiety and preterm birth prediction. *Eur J Obstet Gynecol Reprod Biol* 2019: **237**; 106-112.

Iwanaga R, Sugita N, Hirano E, Sasahara J, Kikuchi A, Tanaka K, and Yoshie H. FcγRIIB polymorphisms, periodontitis and preterm birth in Japanese pregnant women. *Journal of Periodontal Research* 2011: **46**; 292-302.

Javorski N, Lima CAD, Silva LVC, Crovella S, and De Azêvedo Silva J. Vitamin D receptor (VDR) polymorphisms are associated to spontaneous preterm birth and maternal aspects. *Gene* 2018: **642**; 58-63.

Jones NM, Holzman C, Tian Y, Witkin SS, Genc M, Friderici K, Fisher R, Sezen D, Babula O, Jernigan KA, *et al.* Innate immune system gene polymorphisms in maternal and child genotype and risk of preterm delivery. *The Journal of Maternal-Fetal & Neonatal Medicine* 2012: **25**; 240-247.

Kadivnik M, Kralik K, Muller-Vranješ A, Vučemilović-Jurić V, Šijanović S, and Wagner J. Progesterone receptor genetic variants in pregnant women and fetuses as possible predictors of spontaneous premature birth: A preliminary case–control study. *Journal of Obstetrics and Gynaecology Research* 2022.

Karakaş NM, Ecevit AN, Yalçın Y, Özdemir B, Verdi H, Tekindal MA, Özbek NY, Tarcan A, Ataç FB, and Haberal A. Effect of maternal and neonatal interleukin-6 − 174 G/C polymorphism on preterm birth and neonatal morbidity. *The Journal of Maternal-Fetal & Neonatal Medicine* 2018: **31**; 1009-1015.

Karjalainen MK, Huusko JM, Tuohimaa A, Luukkonen A, Haataja R, and Hallman M. A study of collectin genes in spontaneous preterm birth reveals an association with a common surfactant protein D gene polymorphism. *Pediatric Research* 2012a: **71**; 93-99.

Karjalainen MK, Huusko JM, Ulvila J, Sotkasiira J, Luukkonen A, Teramo K, Plunkett J, Anttila V, Palotie A, Haataja R, *et al.* A Potential Novel Spontaneous Preterm Birth Gene, AR, Identified by Linkage and Association Analysis of X Chromosomal Markers. *PLoS ONE* 2012b: **7**; e51378.

Karody VR, Le M, Nelson S, Meskin K, Klemm S, Simpson P, Hines R, and Sampath V. A TIR domain receptor–associated protein (TIRAP) variant SNP (rs8177374) confers protection against premature birth. *Journal of Perinatology* 2013: **33**; 341-346.

Kovac U, Jasper EA, Smith CJ, Baer RJ, Bedell B, Donovan BM, Weathers N, Prosenc Zmrzljak U, Jelliffe-Pawlowski LL, Rozman D, *et al.* The Association of Polymorphisms in Circadian Clock and Lipid Metabolism Genes With 2nd Trimester Lipid Levels and Preterm Birth. *Frontiers in Genetics* 2019: **10**.

Krueger M, Heinzmann A, Mailaparambil B, Härtel C, and Göpel W. Polymorphisms of interleukin 18 in the genetics of preterm birth and bronchopulmonary dysplasia. *Arch Dis Child Fetal Neonatal Ed* 2011: **96**; F299-300.

Kwon BN, Lee NR, Kim HJ, Kang YD, Kim JS, Park JW, and Jin HJ. Folate metabolizing gene polymorphisms and genetic vulnerability to preterm birth in Korean women. *Genes & Genomics* 2021.

Kwon H-S, Sohn I-S, Lee J-Y, Lee S-J, Kim S-N, and Kim B-J. Intercellular adhesion molecule-1 K469E polymorphism in Korean patients with spontaneous preterm delivery. *International Journal of Gynecology & Obstetrics* 2009: **104**; 37-39.

Langmia IM, Apalasamy YD, Omar SZ, and Mohamed Z. Association of VEGFA gene polymorphisms and VEGFA plasma levels with spontaneous preterm birth. *Pharmacogenet Genomics* 2015a: **25**; 199-204.

Langmia IM, Apalasamy YD, Omar SZ, and Mohamed Z. Interleukin 1 receptor type 2 gene polymorphism is associated with reduced risk of preterm birth. *The Journal of Maternal-Fetal & Neonatal Medicine* 2015b; 1-4.

Langmia IM, Apalasamy YD, Omar SZ, and Mohamed Z. Progesterone Receptor (PGR) gene polymorphism is associated with susceptibility to preterm birth. *BMC Medical Genetics* 2015c: **16**.

Langmia IM, Apalasamy YD, Suki SZ, Omar SZ, and Mohamed Z. Genetic association of AKAP10 gene polymorphism with reduced risk of preterm birth. *Journal of Perinatology* 2015d: **35**; 700-704.

Liu G-J, He J-R, Kuang Y-S, Fan X-J, Li W-D, Lu J-H, Xia X-Y, Liu X-D, Chen N-N, Mai W-B, *et al.* Associations of maternal PLA2G4C and PLA2G4D polymorphisms with the risk of spontaneous preterm birth in a Chinese population. *Molecular Medicine Reports* 2017: **15**; 3607-3614.

Liu X, Wang G, Hong X, Tsai H-J, Liu R, Zhang S, Wang H, Pearson C, Ortiz K, Wang D, *et al.* Associations between gene polymorphisms in fatty acid metabolism pathway and preterm delivery in a US urban black population. *Human Genetics* 2012: **131**; 341-351.

Lorenz E, Hallman M, Marttila R, Haataja R, and Schwartz DA. Association between the Asp299Gly Polymorphisms in the Toll-like Receptor 4 and Premature Births in the Finnish Population. *Pediatric Research* 2002: **52**; 373-376.

Lyubomirskaya ES, Kamyshnyi AM, Krut YY, Smiianov VA, Fedoniuk LY, Romanyuk LB, Kravets NY, and Mochulska OM. SNPs and transcriptional activity of genes of innate and adaptive immunity at the maternal-fetal interface in woman with preterm labour, associated with preterm premature rupture of membranes. *Wiad Lek* 2020a: **73**; 25-30.

Lyubomirskaya K, Krut Y, Sergeyeva L, Khmil S, Lototska O, Petrenko N, and Kamyshnyi A. Preterm premature rupture of membranes: prediction of risks in women of Zaporizhzhia region of Ukraine. *Pol Merkur Lekarski* 2020b: **48**; 399-405.

Macones GA, Parry S, Elkousy M, Clothier B, Ural SH, and Strauss JF, 3rd. A polymorphism in the promoter region of TNF and bacterial vaginosis: preliminary evidence of gene-environment

interaction in the etiology of spontaneous preterm birth. *Am J Obstet Gynecol* 2004: **190**; 1504-1508; discussion 1503A.

Manuck TA, Major HD, Varner MW, Chettier R, Nelson L, and Esplin MS. Progesterone Receptor Genotype, Family History, and Spontaneous Preterm Birth. *Obstetrics & Gynecology* 2010: **115**.

Manzon L, Altarescu G, Tevet A, Schimmel MS, Elstein D, Samueloff A, and Grisaru-Granovsky S. Vitamin D receptor polymorphism FokI is associated with spontaneous idiopathic preterm birth in an Israeli population. *Eur J Obstet Gynecol Reprod Biol* 2014: **177**; 84-88.

Marrs C, Chesmore K, Menon R, and Williams S. Maternal human telomerase reverse transcriptase variants are associated with preterm labor and preterm premature rupture of membranes. *PLoS ONE* 2018: **13**; e0195963.

McElroy JJ, Gutman CE, Shaffer CM, Busch TD, Puttonen H, Teramo K, Murray JC, Hallman M, and Muglia LJ. Maternal coding variants in complement receptor 1 and spontaneous idiopathic preterm birth. *Human Genetics* 2013: **132**; 935-942.

Meirhaeghe A, Boreham CAG, Murray LJ, Richard F, Smith GD, Young IS, and Amouyel P. A Possible Role for the PPARG Pro12Ala Polymorphism in Preterm Birth. *Diabetes* 2007: **56**; 494-498.

Menon R, Velez DR, Simhan H, Ryckman K, Jiang L, Thorsen P, Vogel I, Jacobsson B, Merialdi M, Williams SM, *et al.* Multilocus interactions at maternal tumor necrosis factor-alpha, tumor necrosis factor receptors, interleukin-6 and interleukin-6 receptor genes predict spontaneous preterm labor in European-American women. *Am J Obstet Gynecol* 2006a: **194**; 1616-1624.

Menon R, Velez DR, Thorsen P, Vogel I, Jacobsson B, Williams SM, and Fortunato SJ. Ethnic differences in key candidate genes for spontaneous preterm birth: TNF-alpha and its receptors. *Hum Hered* 2006b: **62**; 107-118.

Nan Y and Li H. MTHFR genetic polymorphism increases the risk of preterm delivery. *Int J Clin Exp Pathol* 2015: **8**; 7397-7402.

Pandey M and Awasthi S. Role of MMP-1, MMP-8 and MMP-9 gene polymorphisms in preterm birth. *J Genet* 2020: **99**.

Pandey M, Awasthi S, Singh U, and Mahdi AA. Association of IL-10 Gene Polymorphism (−819C > T, −592C > A and −1082G > A) with Preterm Birth. *The Indian Journal of Pediatrics* 2018: **85**; 93-101.

Pereyra S, Bertoni B, and Sapiro R. Interactions between environmental factors and maternal–fetal genetic variations: strategies to elucidate risks of preterm birth. *European Journal of Obstetrics & Gynecology and Reproductive Biology* 2016: **202**; 20-25.

Plunkett J, Doniger S, Morgan T, Haataja R, Hallman M, Puttonen H, Menon R, Kuczynski E, Norwitz E, Snegovskikh V, *et al.* Primate-specific evolution of noncoding element insertion into PLA2G4Cand human preterm birth. *BMC Medical Genomics* 2010: **3**; 62.

Plunkett J, Doniger S, Orabona G, Morgan T, Haataja R, Hallman M, Puttonen H, Menon R, Kuczynski E, Norwitz E, *et al.* An Evolutionary Genomic Approach to Identify Genes Involved in Human Birth Timing. *PLoS Genetics* 2011: **7**; e1001365.

Preda A, Caracostea G, Ona D, Zaharie G, and Stamatian F. Association between maternal/newborn genetic variants, placental pathology and spontaneous preterm birth risk: a Romanian population-based study. *The Journal of Maternal-Fetal & Neonatal Medicine* 2020: **33**; 1171-1177.

Ramos BRDA, Mendes ND, Tanikawa AA, Amador MAT, Santos NPCD, Santos SEBD, Castelli EC, Witkin SS, and Silva MGD. Ancestry informative markers and selected single nucleotide polymorphisms in immunoregulatory genes on preterm labor and preterm premature rupture of membranes: a case control study. *BMC Pregnancy and Childbirth* 2016: **16**.

Roberts AK, Monzon-Bordonaba F, Van Deerlin PG, Holder J, Macones GA, Morgan MA, Strauss JF, 3rd, and Parry S. Association of polymorphism within the promoter of the tumor necrosis factor alpha gene with increased risk of preterm premature rupture of the fetal membranes. *Am J Obstet Gynecol* 1999: **180**; 1297-1302.

Rocha FG, Slavin TP, Li D, Tiirikainen MI, and Bryant-Greenwood GD. Genetic associations of relaxin: preterm birth and premature rupture of fetal membranes. *American Journal of Obstetrics and Gynecology* 2013: **209**; 258.e251-258.e258.

Romero R, Friel LA, Velez Edwards DR, Kusanovic JP, Hassan SS, Mazaki-Tovi S, Vaisbuch E, Kim CJ, Erez O, Chaiworapongsa T, *et al.* A genetic association study of maternal and fetal candidate genes that predispose to preterm prelabor rupture of membranes (PROM). *American Journal of Obstetrics and Gynecology* 2010a: **203**; 361.e361-361.e330.

Romero R, Velez Edwards DR, Kusanovic JP, Hassan SS, Mazaki-Tovi S, Vaisbuch E, Kim CJ, Chaiworapongsa T, Pearce BD, Friel LA, *et al.* Identification of fetal and maternal single nucleotide polymorphisms in candidate genes that predispose to spontaneous preterm labor with intact membranes. *American Journal of Obstetrics and Gynecology* 2010b: **202**; 431.e431-431.e434.

Rosenfeld T, Salem H, Altarescu G, Grisaru-Granovsky S, Tevet A, and Birk R. Maternal–fetal vitamin D receptor polymorphisms significantly associated with preterm birth. *Archives of Gynecology and Obstetrics* 2017: **296**; 215-222.

Ryckman KK, Morken N-H, White MJ, Velez DR, Menon R, Fortunato SJ, Magnus P, Williams SM, and Jacobsson B. Maternal and Fetal Genetic Associations of PTGER3 and PON1 with Preterm Birth. *PLoS ONE* 2010: **5**; e9040.

Salem H, Rosenfeld T, Altarescu G, Grisaru-Granovsky S, and Birk R. Maternal and neonatal leptin and leptin receptor polymorphisms associated with preterm birth. *Gene* 2016: **591**; 209-213.

Sata F, Toya S, Yamada H, Suzuki K, Saijo Y, Yamazaki A, Minakami H, and Kishi R. Proinflammatory cytokine polymorphisms and the risk of preterm birth and low birthweight in a Japanese population. *Molecular Human Reproduction* 2008: **15**; 121-130.

Schmid M, Grimm C, Leipold H, Knöfler M, Haslinger P, and Egarter C. A polymorphism of the corticotropin-releasing hormone receptor 2 (CRHR2) and preterm birth. *Dis Markers* 2010: **28**; 37-42.

Shim S-Y, Jeong HJ, Park HJ, Kwon EY, Kim BM, Choi YJ, Choi Y-H, Cho SJ, Choi JH, and Park EA. Functional variation of SHP-2 promoter is associated with preterm birth and delayed myelination and motor development in preterm infants. *Scientific Reports* 2017: **7**.

Silva LVCD, Javorski N, André Cavalcanti Brandão L, Lima MDC, Crovella S, and Eickmann SH. Influence of MBL2 and NOS3 polymorphisms on spontaneous preterm birth in North East Brazil: genetics and preterm birth. *The Journal of Maternal-Fetal & Neonatal Medicine* 2020: **33**; 127-135.

Simhan HN, Krohn MA, Roberts JM, Zeevi A, and Caritis SN. Interleukin-6 promoter -174 polymorphism and spontaneous preterm birth. *Am J Obstet Gynecol* 2003: **189**; 915-918.

Song J, Li J, Liu H, Gan Y, Sun Y, Yu M, Zhang Y, Luo F, Tian Y, Wang W, *et al.* A genetic variant in the placenta-derived MHC class I chain-related gene A increases the risk of preterm birth in a Chinese population. *Human Genetics* 2017: **136**; 1375-1384.

Speer EM, Gentile DA, Zeevi A, Pillage G, Huo D, and Skoner DP. Role of Single Nucleotide Polymorphisms of Cytokine Genes in Spontaneous Preterm Delivery. *Human Immunology* 2006: **67**; 915-923.

Stelzer G, Rosen N, Plaschkes I, Zimmerman S, Twik M, Fishilevich S, Stein TI, Nudel R, Lieder I, Mazor Y, *et al.* The GeneCards Suite: From Gene Data Mining to Disease Genome Sequence Analyses. *Current Protocols in Bioinformatics* 2016: **54**.

Thota C, Menon R, Wentz MJ, Fortunato SJ, Bartlett J, Drobek CO, Nair S, and Al-Hendy A. A Single-Nucleotide Polymorphism in the Fetal Catechol-O-methyltransferase Gene is Associated With Spontaneous Preterm Birth in African Americans. *Reproductive Sciences* 2012: **19**; 135-142.

Tiwari D, Bose PD, Das S, Das CR, Datta R, and Bose S. MTHFR (C677T) polymorphism and PR (PROGINS) mutation as genetic factors for preterm delivery, fetal death and low birth weight: A Northeast Indian population based study. *Meta Gene* 2015: **3**; 31-42.

Valdez LL, Quintero A, Garcia E, Olivares N, Celis A, Rivas F, and Rivas F. Thrombophilic polymorphisms in preterm delivery. *Blood Cells, Molecules, and Diseases* 2004: **33**; 51-56.

Velez DR, Fortunato S, Thorsen P, Lombardi SJ, Williams SM, and Menon R. Spontaneous preterm birth in African Americans is associated with infection and inflammatory response gene variants. *American Journal of Obstetrics and Gynecology* 2009: **200**; 209.e201-209.e227.

Velez DR, Fortunato SJ, Thorsen P, Lombardi SJ, Williams SM, and Menon R. Preterm Birth in Caucasians Is Associated with Coagulation and Inflammation Pathway Gene Variants. *PLoS ONE* 2008a: **3**; e3283.

Velez DR, Fortunato SJ, Williams SM, and Menon R. Interleukin-6 (IL-6) and receptor (IL6-R) gene haplotypes associate with amniotic fluid protein concentrations in preterm birth. *Human Molecular Genetics* 2008b: **17**; 1619-1630.

Velez DR, Menon R, Thorsen P, Jiang L, Simhan H, Morgan N, Fortunato SJ, and Williams SM. Ethnic differences in interleukin 6 (IL-6) and IL6 receptor genes in spontaneous preterm birth and effects on amniotic fluid protein levels. *Annals of Human Genetics* 2007: **71**; 586-600.

Vogel I, Hollegaard MV, Hougaard DM, Thorsen P, and Grove J. Polymorphisms in the promoter region of relaxin-2 and preterm birth: involvement of relaxin in the etiology of preterm birth. *In Vivo* 2009: **23**; 1005-1009.

Wang H, Ogawa M, Wood JR, Bartolomei MS, Sammel MD, Kusanovic JP, Walsh SW, Romero R, and Strauss JF. Genetic and epigenetic mechanisms combine to control MMP1 expression and its association with preterm premature rupture of membranes. *Human Molecular Genetics* 2008: **17**; 1087-1096.

Wang H, Parry S, Macones G, Sammel MD, Kuivaniemi H, Tromp G, Argyropoulos G, Halder I, Shriver MD, Romero R, *et al.* A functional SNP in the promoter of the SERPINH1 gene increases risk of preterm premature rupture of membranes in African Americans. *Proceedings of the National Academy of Sciences* 2006: **103**; 13463-13467.

Wang S, Xin X, Luo W, Mo M, Si S, Shao B, Shen Y, Cheng H, and Yu Y. Association of vitamin D and gene variants in the vitamin D metabolic pathway with preterm birth. *Nutrition* 2021: **89**; 111349.

Wang Y, Yang X, Zheng Y, Wu Z-H, Zhang X-A, Li Q-P, He X-Y, Wang C-Z, and Feng Z-C. The SEPS1 G-105A Polymorphism Is Associated with Risk of Spontaneous Preterm Birth in a Chinese Population. *PLoS ONE* 2013: **8**; e65657.

Wang Y, Zhang X-A, Yang X, Wu Z-H, and Feng Z-C. A MCP-1 promoter polymorphism at G-2518A is associated with spontaneous preterm birth. *Molecular Genetics and Genomics* 2015: **290**; 289-296.

Yılmaz Y, Verdi H, Taneri A, Yazıcı AC, Ecevit AN, Karakaş NM, Tarcan A, Haberal A, Ozbek N, and Atac FB. Maternal–Fetal Proinflammatory Cytokine Gene Polymorphism and Preterm Birth. *DNA and Cell Biology* 2012: **31**; 92-97.

Yu Y, Tsai H-J, Liu X, Mestan K, Zhang S, Pearson C, Ortiz K, Xu X, Zuckerman B, and Wang X. The joint association between F5 gene polymorphisms and maternal smoking during pregnancy on preterm delivery. *Human Genetics* 2009: **124**; 659-668.

Zhu Q, Chen Y, Dai J, Wang B, Liu M, Wang Y, Tao J, and Li H. Methylenetetrahydrofolate reductase polymorphisms at 3'-untranslated region are associated with susceptibility to preterm birth. *Transl Pediatr* 2015: **4**; 57-62.
